# Attitudes and intended behaviour to mental disorders and associated factors in catalan population, Spain: cross-sectional population-based survey

**DOI:** 10.1186/s12889-016-2815-5

**Published:** 2016-02-09

**Authors:** Ignacio Aznar-Lou, Antoni Serrano-Blanco, Ana Fernández, Juan V. Luciano, Maria Rubio-Valera

**Affiliations:** 1 Teaching, Research & Innovation Unit, Fundació Sant Joan de Déu, Esplugues de Llobregat, Spain; 2 Primary Care Prevention and Health Promotion Research Network (redIAPP), Barcelona, Spain; 3 Mental Health Policy Unit, The Brain and Mind Research Institute; and Centre for Disability Research and Policy, Faculty of Health Sciences, University of Sydney, Sydney, Australia; 4 School of Pharmacy, University of Barcelona, Barcelona, Spain

**Keywords:** Social stigma, Mental-Health, Social Discrimination, Spain

## Abstract

**Background:**

Mental disorders have a huge impact on the European population. Two of the main causes of this impact are stigma and discrimination. The aim of this paper is to assess the stigma regarding mental disorder in Catalonia and to explore factors associated with stigma.

**Methods:**

Cross-sectional population-based survey of a representative sample of non-institutionalized adult population (*n* = 1872). We evaluated attitudes (CAMI: Authoritarianism, Benevolence and Support to Community Mental Health care) and intended behaviour (RIBS) regarding mental disorder and experience of discrimination. Higher scores showed more favourable attitudes and intended behavior. Mean values and percentiles of the scales were calculated. Multivariable regression models were used to assess factors associated with stigma.

**Results:**

Mean authoritarianism, benevolence and support to community mental health scores corresponded to the 66th, 90th and 78th percentile, respectively. Mean RIBS score corresponded to the 76th percentile. More favourable attitudes were associated with being male, younger, having a higher education, being Spanish, having suffered a mental disorder and having contact with a person with a mental disorder.Similarly, more favourable intended behaviour was associated with being younger, having secondary education, having Spanish nationality, belonging to a higher social class and having contact with a person with a mental disorder. People with depression or anxiety showed lower discrimination experiences than people with other mental disorders.

**Conclusions:**

The levels of stigma were generally low among the Catalan population. However, efforts should be made to decrease stigma related to authoritarianism. Interventions addressed to reducing stigma should take into account other mental disorders apart from depression or anxiety. They should be focused on older, immigrant population, people with lower educational attainment and people who have not had contact with someone with a mental disorder.

## Background

Mental Disorders (MDs) have a huge impact on the European population. It is estimated that between 9 and 38 % of Europeans experience a MD every year [1, 2] and between 10 and 16 % of global Disability Adjusted Life-Years in western Europe can be attributed to MDs. People with mental disorder (MD) are also victims of stigma and discrimination, which negatively impact their health and social outcomes [3].Stigma is associated with social isolation, delayed help-seeking and loneliness [4].Furthermore, stigma not only impacts the people who suffer a MD but also their relatives and caregivers; for whom it is a potent source of distress [5].

Stigma is a multidimensional phenomenon [6]that comprises a cognitive and a behavioural construct [7].The cognitive construct is, in turn, made of two basic constructs: stereotypes (social knowledge of a specific group) and prejudice (the attitude toward the group involved). The behavioural construct, discrimination, is the result of the cognitive construct, ie, the behavioural consequences of stereotypes and prejudice [7]. However, other conceptualizations of stigma exist and widely varying instruments have been employed to measure the distinct constructs of stigma [8],which hampers comparisons between studies evaluating the levels of public stigma [9]. Scales such as the Community Attitude towards the Mentally Ill scale (CAMI) and the Depression Stigma Scale (DSS) assess the respondent stereotypes and prejudices in dimensions such as authoritarianism, benevolence and social restrictiveness [10, 11]. Measures of social distance, such as the Reported and Intended Behaviour Scale (RIBS), seek to assess the respondent’s willingness to interact with a target person in different types of relationships. However, behavioural responses may differ from reported intentions and measures of experienced discrimination have been employed to assess discriminatory behaviour.

Despite efforts to improve public knowledge and attitudes and reduce discrimination, public stigma is still high [9]. A European study (in Germany, Ireland, Portugal and Hungary) using the DSS showed a moderate degree of personal stigma toward depression and a high degree of perceived stigma [12]. Similar studies developed in Germany and France showed that more than one in four people think that a person who suffers schizophrenia is dangerous. This rate increases even more when people are questioned about their feelings of fear towards people who have schizophrenia [13, 14]. Experience of discrimination and social exclusion due to mental illness in European countries is not insignificant. European studies showed that more than half of the subjects who had a schizophrenic disorder or were an inpatient in a mental healthcare centre referred to having been discriminated against because of the MD [15–17]. Studies developed in non-European countries showed that mental illness is also highly stigmatized in Asia [18, 19], Africa [20] and Latin-America [21].

Between 2009 and 2012, attitudes and intended behaviour of the general population in England were evaluated through CAMI and RIBS scales. In 2012, attitudes towards the mentally ill were moderate-good, with a 78 % of the population agreeing to positive items of the CAMI, while around 71.2 % of the population was willing to interact with people with a mental illness [22].

The literature shows some factors associated with stigma toward people who suffer a MD. A study showed that being female, younger and white, having a higher social class, having a MD and being in contact with a person with MD were associated with more favourable attitudes and/or intended behaviour to MD [22]. In a study developed in four European countries, female gender and younger age were associated with more positive attitudes, showing these studies have similar findings. In addition, this study also showed living alone and lower education level as factors associated with more negative attitudes [12].

Little is known about the levels of stigma and factors associated with attitudes and intended behaviour in the Spanish population. A study conducted in the autonomous community of Madrid found significant associations between higher stigma (measured with the Attribution Questionnaire-27) and older age, being married, living in the city of Madrid (in contrast with those living in the metropolitan area) and having lower knowledge about the MD [23].However, the study was carried out in a small (*n* = 439) non-representative sample of the population that was selected from the places of residence of the researchers team.

There is little evidence of stigma levels associated with MDs in Southern European countries. Studies in this area have been focused on specific diseases such as schizophrenia. To the best of our knowledge, this is the first study which evaluates stigma levels toward non-specific MDs in a representative sample of the population in Catalonia, region located in Spain.

The aims of the present study were (1) to assess the level of attitudes and intended behaviour regarding mental health among a representative sample of the general population in Catalonia (Spain) and (2) to explore factors associated with better attitudes and intended behaviour.

## Methods

### Study design

We conducted a cross-sectional population-based survey. The survey was included in an ongoing wave of the Catalan Health Survey (Enquesta de Salut de Catalunya, ESCA). The ESCA has been conducted twice-yearly by the Department of Health of the Government of Catalonia since 1994 to assess the overall health status, life style and use of health services of the Catalan population. ESCA is an official survey that meets all the Spanish regulatory requirements including data confidentiality. All participants provided informed consent [24, 25].

### Participants

ESCA is a household interview survey of a multistage probability sample representative of the non-institutionalized residents of Catalonia. It has no exclusion criteria apart from being older than 14 years of age. From 2010-14, ESCA was structured in eight biannual stages of approximately 2,400 interviews each [26]. The sample included in this article were survey respondents who were interviewed between July and December 2013 (*n* = 1,872). The sampling strategy was stratified (by territory, gender and age). The basic territorial units were the area health authorities. Consequently territories were stratified under five categories according to their population size and, then, randomly selected within the 37 area health authorities. Territories with lower population density were overrepresented. Afterwards, individuals from the selected territories were classified by gender and age into 13 groups. Random sample selection of the participants (each with 10 substitutes) were made from each gender and age stratum using SAS. A probability weight based on the sampling strategy was calculated.

### Measures

#### Sociodemographic and clinical characteristics

Sociodemographic characteristics collected were gender, age, education level, social class and nationality. Social class was based on occupation with three categories: high, medium and low [26]. High social class is composed of managerial, administrative and professional occupations; medium social class is composed by qualified workers; and low social class is composed of semi-qualified and non-qualified workers. This classification is based on the Official Social Classification in the United Kingdom [27]. Nationality was treated as a categorical variable: Spanish, European, American, African or Asian-Oceanian. People with dual nationality (including Spanish) were treated as Spaniards. Contact with MD(close relative, other relative, friend or other person) was also measured. Experiences of discrimination, including discrimination because of a MD, were evaluated through a discrimination experiences scale in various situations [28, 29]. Having had depression or anxiety and/or another MD was evaluated as a self-reported clinical characteristic and we consider it as a clinical variable.

#### Attitudes

A Spanish 23-item version of the Community Attitudes Towards the Mentally Ill scale (CAMI-23) was used to measure mental-health related attitudes [10, 30]. The CAMI items are rated on a five-point Likert-type scale from 1 (strongly agree) to 5 (strongly disagree). Scores of postitive items were reverse coded so that higher scores indicate more favorable attitudes. In order to avoid excess of data and ease data interpretation in the descriptive analyses, CAMI items were dichotomized as in previous study [22]: scores 1 to 3 were considered as people who disagree or are neutral while score 4 and 5 were considered people who agree. Then, CAMI items were distributed into 3 subscales based on a factor analysis conducted in a previous study [30]: Authoritarianism (7 items), Benevolence (6 items) and Support for community mental health care (SCMH) (10 items). The Spanish version has sound psychometric properties [30].

#### Intended behaviour

Measuring behaviour is complex because an evaluation of the entire process of behaviour change would be needed. In order to facilitate the measurement of behavior, proxy measures, such as intended behaviour, have been developed [8].

In this study RIBS was used to assess the intended behaviour in relation to future contact with people with mental health problems (intention to live with, work with, live nearby and continue a relationship with someone with a mental health problem) [31]. The four items of the scale are rated on a five-point Likert scale from 1 (strongly disagree) to 5 (strongly agree) so that higher scores indicate more favorable intended behaviour. RIBS items were dichotomized so that 4–5 scores were considered people who agree with the item. The total score of the RIBS showed good reliability (α = 0.89).

### Statistical analysis

Stata 13 for Windows was employed to conduct all the analyses.

An inverse probability weight was applied to correct overrepresented groups of the population due to the sampling strategy of the ESCA. The variables with missing data were imputed. The proportion of missing values in the items of the CAMI and RIBS ranged from 1.82 to 35.95 %, however, only 3 items had a proportion of missing values higher than 10 %. Multiple imputation was used under a missing at random assumption in order to impute the missing values. The method used was predictive mean matching [32, 33].In the imputation all available covariates were included in the regression and 200 imputed datasets were created. All the analysis involving CAMI and RIBS scales were conducted in each of the imputed databases. Rubin’s rules were used to combine point and variance estimates from the multiply-imputed datasets [33].

### Levels of stigma and associated factors

We calculated descriptive analyses for sociodemographic and clinical characteristics. The proportion of population with contact with MD and who had experienced discrimination because of a MD were calculated. The levels of stigma (attitudes and intended discrimination) in the general Catalan population were calculated as the mean value of the three CAMI subscales (authoritarianism, benevolence and SCMH) and RIBS scale. Additionally, subscales mean scores were shown as percentile scores to allow for comparison between subscales. To construct these percentile scores, the difference between the mean subscale score obtained and the minimum possible score (5 for all subscales) was calculated. This result was then divided for the corrected maximum possible subscale score (30 for authoritarianism, 25 for benevolence, 45 for SCMH and 15 for Intended behaviour). Also, the proportion of the population agreeing with each of the CAMI and RIBS items was calculated.

To evaluate the factors associated with attitudes and intended behavior, first, bivariate linear regression was used with CAMI and RIBS scales as the dependent variables, and sociodemographic and clinical variables as independent variables. We tested those variables that had been reported in the past to be associated with stigma or that we suspected could have an impact on stigma. Age was categorized to present mean attitudes and intended behaviour in different age groups. The results of the bivariate analyses were used to decide which variables were finally included in the multivariate analyses. All the variables that predicted the dependent variable (*p* < 0.20) [34] in bivariate regression analysis were introduced in a multivariable linear regression model. Interactions between gender, nationality (a dichotomic variable –Spanish vs non-Spanish population– was used because interactions excessively stratified participants), social class and presence of MD with the rest of variables were examined. Statistically significant interactions were reported in multivariate regression models.

## Results

### Sample characteristics

Sample characteristics are presented in Table 1. The sample was composed of 1872 participants. The sample was representative of the Catalan population. The proportion of men and women was very similar and the mean age was 46.8. The sample was composed, mainly, of people with Spanish nationality (89.1 %). The majority of the subjects had secondary studies (61.0 %) and belonged to medium social class (53.1 %). One in five people reported having had a MD; most of these disorders were depression or anxiety disorders.

**Table 1:** Sociodemographic and clinical characteristics of the sample

	*n* = 1872	%	95 % CI
Gender			
Men	949	50.5	47.1; 52.0
Women	923	49.5	48.0; 52.9
Age (mean = 46.80; SE = 0.45)			
15–44	911	48.7	47.3; 52.2
45–64	572	30.6	28.8; 33.3
65–74	183	9.8	8.6; 11.6
≥75	206	11.0	7.8; 10.4
Nationality			
Spaniards	1,667	89.1	87.6; 90.6
European (not Spain)	57	3.0	2.2; 3.8
African	58	3.1	2.2;3.9
American	82	4.4	3.3;5.4
Asian/Oceanian	8	0.4	0.1;0.7
Education level			
Primary	356	19.9	18.0; 21.8
Secondary	1,176	60.9	58.6; 63.3
University	336	19.1	17.1; 21.0
Not answered	1	0.1	-0.1;0.2
Social Class (Occupation)			
Low	446	23.9	21.8; 26.0
Medium	1,002	53.1	50.6; 55.5
High	351	19.3	17.3; 21.2
Not known/answered	73	3.7	2.8; 4.7
MD Experience			
Any MD^a^	340	19.2	17.3; 21.1
D/A	326	18.4	16.4; 20.3
Other MD	26	1.5	0.9; 2.2
Contact with MD			
Any contact	873	48.9	46.4; 51.3
Close relative	338	18.6	16.7;20.5
Other relative	252	14.6	12.9; 16.4
Friend	308	18.0	16.1; 19.9
Other person	363	20.3	18.3; 22.2
With any MD	227	65.2^b^	59.8; 70.6
With D/A	218	65.1^b^	59.6; 70.7
Other MD	18	67.5^b^	47.4; 87.7
Discriminated because of MD			
All population^c^	12	0.8	0.3; 1.3
With D/A	10	3.8 ^b^	1.4; 6.1
With other MD	4	15.0 ^b^	0.1; 29.9

*MD* Mental Disorder, *D/A* Depression or Anxiety

^a^Some subjects referred to have Depression or Anxiety and other Mental Disorder

^b^These rates are in relation with the total number of subjects of the specified item

^c^Some subjects referred to have been discriminated because of Depression or Anxiety and other Mental Disorder

### Contact with MD and discrimination

Around half of the subjects of the sample had been in contact with a person with a MD. Among people with a MD, the proportion of subjects discriminated against because of a MD was 3.8 % in people with depression or anxiety disorder, and 15.0 % in people who had another MD. In total, these rates represented less than1% of the overall population experiencing discrimination because of a MD.

### Attitudes and intended behaviour in the general population

Attitudes (CAMI) and intended behaviour (RIBS) in the general population are presented in Table 2. Authoritarianism, benevolence and SCMH mean scores were 24.9, 27.6 and 40.2 respectively. These mean scores correspond to the 66th, 90th and 78th percentile, respectively, underlining that participants showed more favourable attitudes in the benevolence and SCMH subscales than in the authoritarianism scale.

**Table 2:** Mean CAMI and RIBS scales scores (with percentile) and proportion of population that agrees with them

Scale CAMI	Mean value or proportion (%)	IC 95 %
Mean value (and decile score) for the Authoritarianism subscale	24.9 (66.3)	24.7; 25.1
Proportion of population that agree to the Authoritarianism subscale items		
1. One of the main causes of mental illness is a lack of self-discipline and will-power^a^	53.2	50.7; 55.8
2. There is something about people with mental illness that makes it easy to tell them from normal people^a^	39.0	36.5; 41.4
3. As soon as a person shows signs of mental disturbance, he should be hospitalized^a^	52.1	49.6; 54.6
4. Mental hospitals are an outdated means of treating people with mental illness	22.3	19.9; 24.6
5. People with mental illness are a burden on society^a^	75.1	73.0; 77.2
6. People with mental illness should not be given any responsibility^a^	51.2	48.7; 53.7
7. Anyone with a history of mental problems should be excluded from taking public office^a^	72.5	70.2; 74.7
Mean value (decile) for the Benevolence subscale	27.6 (90.4)	27.5; 27.7
Proportion of population that agree to the Benevolence subscale items		
1. Virtually anyone can become mentally ill	92.2	90.9; 93.6
2. People with mental illness have for too long been the subject of ridicule	74.7	72.5; 76.9
3. We need to adopt a far more tolerant attitude toward people with mental illness in our society	91.8	90.4; 93.2
4. We have a responsibility to provide the best possible care for people with mental illness	93.7	92.5; 94.9
5. People with mental illness don’t deserve our sympathy^a^	95.3	94.2; 96.3
6. Increased spending on mental health services is a waste of money^a^	93.8	92.6; 95.0
Mean value (decile) for the SCMH subscale	40.2 (78.2)	39.9; 40.5
Proportion of population that agree to the SCMH subscale items		
1. I would not want to live next door to someone who has been mentally ill^a^	71.4	69.2; 73.7
2. No-one has the right to exclude people with mental illness from their neighbourhood	84.1	82.3; 85.9
3. People with mental illness are far less of a danger than most people suppose	62.0	59.5; 64.5
4. Most people who were once patients in a mental hospital can be trusted to take care of other (babysitters, etc.)	44.2	41.7; 46.8
5. The best therapy for many people with mental illness is to be part of a normal community	85.2	83.3; 87.0
6. As far as possible, mental health services should be provided through community based facilities	73.3	71.1; 75.6
7. Residents have nothing to fear from people coming into their neighborhood to obtain mental health services	74.4	72.1; 76.6
8. It is frightening to think of people with mental problems living in residential neighborhoods^a^	77.6	75.6; 79.7
9. Locating mental health facilities in a residential area downgrades the neighborhood^a^	84.4	82.6; 86.3
Mean value (decile) for the RIBS subscale	16.4 (76.1)	16.2; 16.6
Proportion of population that agree to the RIBS subscale items		
1. In the future, I would be willing to live with someone with a mental health problem	62.9	60.5; 65.3
2. In the future, I would be willing to work with someone with a mental health problem	73.1	70.9; 75.4
3. In the future, I would be willing to live nearby to someone with a mental health problem	74.6	72.4; 76.8
4. In the future, I would be willing to continue a relationship with a friend who developed a mental health problem	79.9	77.9; 82.0

*SCMH* Support for Community Mental Health care

^a^The score of the item was inverted in order to suit with an desirable score

Most people had favourable scores for items of the authoritarianism subscale except item 2 (“There is something about people with mental illness that makes it easy to tell them from normal people”; 39.0 % disagreed) and item 4 (“Mental hospitals are an outdated means of treating people with mental illness”; 22.3 % disagreed).

Most people had favourable scores for items of the SCMH subscale except item 4 (“Most people who were once patients in a mental hospital can be trusted to take care of other people (for example, babysitting, etc.)”; 44.3 % disagreed).

Regarding intended behaviour, the proportion of people agreeing with each of the RIBS item ranged between 63 and 80 % with a mean RIBS score of 16.4 (76th percentile), which shows a moderate-high intention to have contact with people with a MD in the future.

### Factors associated with attitudes and intended behaviour

Table 3 presents attitudes and intended behaviour based on bivariate regression models for subgroups of patients according to sociodemographic and clinical characteristics. The results of the multivariate regression models are presented in Table 4.

**Table 3:** Mean CAMI and RIBS scales (and decile) for sociodemographic and clinical characteristics

Categories	Authoritarianism (range 5–35)	95 % CI	Benevolence (range 5–30)	95 % CI	SCMH (range 5–50)	95 % CI	Intended Behaviour (range 5–20)	95 % CI
Gender								
Men	27.6 (75.4)a	27.5; 27.7	27.5 (90.1)	27.3; 27.7	40.4 (78.7)	40.0; 40.8	16.6 (77.6)a	16.4; 16.9
Woman	25.2 (67.5)a	24.9; 25.6	27.7 (90.8)	27.5; 27.9	40.0 (77.8)	39.6; 40.4	16.2 (76.5)a	15.9; 16.5
Age category								
15–44	25.6 (68.6)3,4	25.3; 25.9	27.5 (90.2)2,4	27.3; 27.7	40.6 (79.0) 3,4	40.2; 41.0	16.9 (79.4)3,4	16.7; 17.1
45–64	25.3 (67.8)3,4	24.9; 25.8	28.0 (92.2)1,4	27.8; 28.3	40.7 (79.4)3,4	40.2; 41.2	16.6 (77.5)3,4	16.3; 17.0
65–74	22.4 (58.0)1,2	21.7; 23.1	27.5 (90.1)4	27.0; 28.0	38.9 (75.3)1,2	37.9; 39.9	15.3 (68.5)1,2,4	14.6; 16.0
>75	22.4 (58.0)1,2	21.7; 23.1	26.5 (86.2)1,2,4	26.0; 27.1	38.0 (73.4)1,2	37.0; 39.0	14.2 (61.3)1,2,3	13.5; 14.9
Education level								
Primary	23.1 (60.2)	22.6; 23.6	26.9 (87.8)	26.6; 27.3	35.7(68.3)	35.0; 36.4	15.3 (68.6)	14.8; 15.8
Secondary	24.8 (66.1)b	24.5;25.1	27.8 (91.0)b	27.5;28.1	37.8 (72.8)b	37.4; 38.1	16.7 (77.9)b	16.5; 16.9
Universitary	27.0 (73.3) b	26.5; 27.5	27.7 (91.3)b	27.6;27.9	38.0 (73.3) b	37.3;38.6	16.7 (78.1) b	16.3; 17.1
Social Class								
Low	24.3 (64.3)c	23.8; 24.7	27.4 (89.6)c	27.1;27.7	37.0 (71.2)	36.4;37.6	16.0 (73.3)c	15.6;16.4
Medium	24.8 (66.1)c	24.5; 25.1	27.6 (90.5)c	27.4;27.8	37.5 (72.2)c	37.1;37.8	16.5 (76.6)c	16.2;16.7
High	26.1 (70.5)	25.6;26.7	28.0 (92.0)	27.7;28.3	38.2 (73.7)	37.6;38.7	17.1 (80.8)	16.7;17.5
Nationality								
Spanish	25.0 (66.8)	24.8; 25.3	27.7 (90.7)	27.5; 27.8	37.5 (72.2)	37.2; 37.8	16.5 (76.7)	16.3;16.7
European (not Spain)	23.9(62.9)	22.6; 25.2	27.8 (91.2)	27.2; 28.4	37.3 (71.8)	35.6; 39.0	16.6 (77.6)	15.7; 17.5
African	23.5 (61.6) d	22.4; 24.6	27.0 (88.2)	26.1; 28.0	36.4 (69.9)	34.7; 38.2	15.5 (69.8)	14.3; 16.6
American	23.9 (62.9) d	22.8; 24.9	26.9 (87.5)	26.0; 27.7	36.2 (69.4)	34.9; 37.6	15.3 (69.0)d	14.4; 16.3
Asian/Oceania	20.9 (53.1)d	15.8; 26.0	25.4 (81.5)d	22.5; 28.3	34.3 (64.8) d	30.1; 38.4	13.7 (57.8)dd	11.8; 15.5
Having had a MD								
With a MD	25.5 (68.2)a	24.9; 26.0	27.9 (91.7)a	27.6; 28.2	40.1 (78.1)	39.4; 40.8	16.4 (76.3)	16.0; 16.9
Without a MD	24.7 (65.8) a	24.5; 25.0	27.5 (90.1)a	27.4; 27.7	40.2 (78.3)	39.9; 40.6	16.4 (76.0)	16.2; 16.6
Contact with MD								
Contact with anyone with a MD	26.1 (70.5)a	25.8; 26.4	28.2 (92.8)a	28.0; 28.4	38.6 (74.6)a	38.2; 38.9	17.4 (82.9) a	17.2; 17.7
No contact with MD	23.7 (62.3)a	23.4; 24.0	27.0 (88.2)a	26.8; 27.3	36.3 (69.5)a	35.9; 36.6	15.4 (69.5) a	15.2; 15.7

^a^ *p* < 0.05: differences between values of each variable in unadjusted regression models

1: Differences (*p* < 0.05) regarding to group 15–44 years old. 2. Differences (*p* < 0.05) regarding to group 45–64 years old. 3. Differences (*p* < 0.05) regarding to group 65–74 years old. 4. Differences (*p* < 0.05) regarding to group older than 75 years old in bivariate ordered logistic regression models

^b^Differences (*p* < 0.05) regarding to population with primary studies (considered as reference category in Education level variable)

^c^Differences (*p* < 0.05) regarding to High Social Class (considered as reference category in Social class variable)

^d^Differences (*p* < 0.05) regarding to Spaniards (considered as reference category in Nationality variable).^dd^Differences (*p* < 0.001) regarding to Spaniards (considered as reference category in Nationality variable)

**Table 4:** Factors associated to attitudes and intended behavior using a multivariate regression model

	Authoritarianism		Benevolence		SCMH		Intended Behaviour	
	β	95 % CI	β	95 % CI	β	95 % CI	β	95 % CI
Constant	24.2	23.0;25.4	26.2	25.4; 26.9	37.1	35.6;38.6	18.0	17.0;19.0
Gender								
Women	ref	-	-	-	ref	-	ref	-
Men	0.7a	0.2; 1.1	-	-	-0.3	-0.8;0.2	-0.4a	-0.8;-0.1
Age (Continuous)	-0.05b	-0.06;-0.03	0.01	0.00;0.01	-0.02a	-0.04;-0.01	-0.03b	-0.04;-0.02
Educational level								
Primary	ref	-	ref	-	ref	-	ref	-
Secondary	0.8a	0.2;1.4	0.8a	0.3;1.2	1.3a	0;5;2.1	0.5a	0.1;1.0
Universitary	2.7b	1.9;3.5	0.6a	0.1;1.1	1.1a	0.1;2.1	0.05	-0.6;0.7
Social Class								
Low	ref	-	ref	-	ref	-	ref	-
Medium	0.03	-0.5;0.6	0.1	-0.2;0.5	0.1	-0.6;0.8	0.3	-0.2;0.8
High	0.2	-0.5;0.9	0.4	-0.1;0.8	0.5	-0.4;1.4	0.9a	0.3;1.5
MD								
Not having suffered it	ref	-	ref	-	-	-	-	-
Having suffered it	0.7a	0.2; 1.3	0.3	-0.1; 0.6	-	-	-	-
Nationality								
Spanish	ref	-	ref	-	ref	-	ref	-
European (not Spain)	-1.3a	0.7;2.1	0.4	-0.3;1.0	-0.1	-0.1;1.7	-0.3	-1.2;0.6
African	-1.2a	-2.3;-0.2	-0.03	-0.9;0.9	-0.5	-2.2;1.3	-1.2 b	-2.4;-0.01
American	-1.4a	-2.4;-0.4	-0.6	-1.4;0.2	-1.3	-2.6;0.01	-1.6a	-2.5;-0.6
Asian/Oceanian	-3.8	-8.1;0.5	-1.5	-3.6;0.6	-2.2	-5.2; 0.8	-3.1b	-4.7;-1.6
Contact with MD								
No	ref	-	ref	-	ref	-	-	-
Yes	1.8b	1.3; 2.2	1.0b	0.7; 1.3	2.0b	1.4; 2.5	1.7 b	1.4-2.1

SCMH Support for Community Mental Health care, β Coefficient

-:Variables with p value >0.20 in bivariate linear regression and, therefore, not included in multivariate linear regression

^a^ *p* < 0.05

^b^ *p* < 0.001

In the bivariate analysis, there were differences according to gender in both authoritarianism and intended behaviour (Table 3). However, after adjustment, it was only retained for the authoritarianism subscale (Table 4). In this subscale, men had a mean score of 27.6 (75th percentile) compared to 25.2 (68th percentile) in women, with more favourables cores for men.

The youngest group (15 to 44 years old) showed the highest score in authoritarianism and intended behaviour while for benevolence and SCMH, people from 45 to 64 years old showed the highest scores. Older people showed worse attitudes and intended behaviour for all the subscales except benevolence.

People with primary education showed worse attitudes and intended behavior for all subscales in bivariate analysis. These differences were maintained in all attitude subscales in the multivariate analysis. However, in this analysis, there are no differences between people who have primary and university education in intended behaviour but the differences in intended behaviour are remained if we contrast people with primary and secondary education.

The only subscale where significant differences were found in social class variable, after performing the multivariate analysis, was intended behavior, with more favourable scores for high social class (17.2; 81st percentile vs 12.5; 77th percentile and 16.0; 73rd percentile for medium and low category).

In bivariate analysis, Asian-Oceanian population showed less favourable attitudes than Spanish population in all subscales; these differences were larger in this population than in the rest of nationalities. In addition, less favorable attitudes were found in people from America and Africa in authoritarianism. Less favorable intended behaviour was found in the American population. However, after adjustment, only authoritarianism and intended behaviour subscales maintained significant differences between groups. In authoritarianism, differences between Spanish and Asian-Oceanian population disappeared despite of the high mean difference (β = -3.8). Differences existed between Spaniards and other European, African and American. In intended behavior, differences were observed between Spaniards and African, American and Asian-Oceania (16.5; 76th percentile, 15.5; 69th percentile, 15.3; 69th percentile and 13.7; 57th percentile, respectively).

Having had a MD was associated with higher scores on authoritarianism and benevolence subscales, but the only subscale which maintained this association after adjustment was authoritarianism, getting higher scores(25.5; 68th percentile vs 24.8; 66th percentile) in people who have had a MD.

Knowing someone with a MD was associated with better attitudes in all subscales and intended behaviour after adjusting in multivariate analysis with a positive strong association (*p* < 0.001).

### Interactions

Table 5 shows the models with statistically significant interactions. In the authoritarianism subscale, social class interacts with education level. Remarkable interaction is the modulation of education level in high social class. People with university education have more favourable attitudes when they are located in high social class regarding to low social class (27.1 vs 22.7). On the other hand population with primary studies has less favourable attitudes independently of social class. The effect of social class in people with primary studies is negative when social class is increased, however this effect is positive in people of high social class. In benevolence subscale we found statistically significative interactions between social class and having a MD, however this interaction is small (the larger difference is 0.6). We found similar situation in the interaction between gender and nationality (the larger difference is 0.8). There are not statistically significative interactions in SCMH subscale. In intended behaviour, we found interactions between age and having a MD and between age and gender; in both interactions younger population had better intended behaviour although coefficient was higher in the interaction with having had a MD (-0.3 vs -0.02).

**Table 5:** Multivariate regression model with significant interaction terms

	Authoritarianism		Benevolence		Intended Behaviour	
	β	95 % CI	β	95 % CI	β	95 % CI
Constant	24.2	22.9;25.5	26.8	25.4; 28.3	15.5	14.4;16.7
Gender						
Women	ref	-	ref*	-	ref	-
Men	0.7^a^	0.3; 1.1	-0.8	-1.7:0.6	-0.1	-1.0;1.2
Age (Continuous)	-0.05^b^	-0.06;-0.03	0.004	-0.004;0.01	-0.01^a^	-0.03;-0.0004
Educational level						
Primary	ref	-	ref	-	ref	-
Secondary	2.2^a^	0.2;1.4	0.8^b^	0.4;1.2	0.5	0.02;1.0
Universitary	4.5^b^	1.9;3.4	0.6^a^	0.1;1.1	-0.04	-0.7;0.6
Social Class						
Low	ref	-	ref	-	ref	-
Medium	-1.7	-3.3;0.002	0.3	-0.1;0.7	0.4	-1.0;1.8
High	-0.8	-3.6;2.1	0.6 ^a^	0.1;1.1	2.2 ^a^	0.6;3.9
MD						
Not having suffered it	ref	-	ref	-	ref	-
Having suffered it	0.7^a^	0.2; 1.3	-0.1	-0.9; 0.6	1.8 ^a^	0.5;3.1
Nationality						
Not Spanish	ref	-	ref	-	ref	-
Spanish	-0.2	-2.2; 1.7	0.2	-0.3;0.7	0.8^a^	0.2;1.4
Contact with MD						
No	ref	-	ref	-	ref	-
Yes	1.8^b^	1.4; 2.2	1.0^b^	0.7; 1.3	1.7^b^	1.4-2.1
Interactions						
Social class x Education level						
Low Social Class x Primary Education	ref	-	-	-	-	-
Medium Social Class x Secondary Education	0.7	-0.5;1.9	-	-	-	-
Medium Social Class x University Education	1.9	-0.3;4.1	-	-	-	-
High Social Class x Secondary Education	1.6	-0.6;3.7	-	-	-	-
High Social Class x University Education	3.4 ^a^	0.7;6.1	-	-	-	-
Social Class x Having a MD						
Low Social Class x having a MD	-	-	ref	-	-	-
Medium Social Class x having a MD	-	-	-0.9 ^a^	-1.7;-0.1	-	-
High Social Class x having a MD	-	-	-1.1^a^	-2.0;-0.9	-	-
Nationality x Gender						
Non Spanish x Woman	-	-	ref	-	-	-
Spanish x Men			1.0^a^	0.03;1.9	-	-
Gender x Age						
Men x Age	-	-	-	-	-0.02 ^a^	-0.04;-0.004
Age x Having a MD						
Age x Having a MD	-	-	-	-	-0.3^a^	-0.1;-0.01

SCMH Support for Community Mental Health care, β Coefficient

-:Variables with *p* value >0.20 in bivariate linear regression and, therefore, not included in multivariate logistic regression. In interactions section: *p* > 0.05 and not included in model/ Interactions with *p* value >0.05 in multivariate linear regression

*An exception is done in gender in benevolence model because gender variable had a *p* value > 0.20, however the variable was included as part of an interaction

^a^ *p* < 0.05 ^b^ *p* < 0.001

h = High; m = Medium; l = Low; p = Primary; s = Secondary; u = University

## Discussion

Stigma (attitudes, intended behaviour and discrimination) were assessed in a representative sample of general non-institutionalized population in Catalonia (Spain) in 2013. Attitudes toward people with mental illness varied among the different constructs of prejudices. The population showed better attitudes in the benevolence and SCMH subscales than in the authoritarianism scale, with very high scores in the benevolence subscale. This is consistent with results of other studies that observed increases in perceived danger through contact with people with MD [10].The level of stigma shown by the Catalan population is, mostly, in line with or better than other European populations [12, 22, 35]. With regard to factors associated with stigma, young people with secondary or university studies who have been in contact with a person with MD are typical of that section of the Catalan population who, as our study shows, have more favourable attitudes towards people with MD. Specifically, other factors such as being Spanish, male and having had a MD feature on the authoritarianism subscale. On the other hand, the profile of people with more favourable intended behaviour is characterized by being Spanish, young, having completed secondary education and belonging to a high social class. Higher scores in secondary or university studies could be explained by higher health literacy [18, 36]. Similarly, people located in a low social class are, also, supposed to have lower knowledge about MD [37]. Difference in nationality could be explained by cultural and ethnic factors, which influence stigma levels [38]. These differences are particularly relevant for benevolence subscale. In this subscale Spanish population have better attitudes independently of gender despite of men showed less favourable attitudes in general population.

This study shows, as some authors have pointed out [39–41], that the use of social class based on occupation as a demographic feature is controversial. Distinctions between different social classes are not sufficiently clear and some authors suggest that education level would be a more precise socioeconomic variable. In our study we observe how differences between education levels are maintained in all subscales in multivariate analysis while social class only shows differences in intended behaviour scale. In line with this, when interactions are tested in authoritarianism subscale we observe education level as the main variable which determines attitudes. Although social class, also, impacts on attitudes, it is pushed into the background.

Less favourable attitudes in authoritarianism and intended behaviour in non-Spanish population could be explained by stigma levels on their country of origin. Stigma levels are moderate-high in countries located in Africa or Asia [18, 20], this is in line with the results of other studies that showed higher levels of stigma among immigrant from Turkey [42] and China [43].

Evans-Lacko and colleagues evaluated the attitudes and intended behaviour of the English population in 2009-2012 [22]. Although difference between benevolence, SCMH subscale and intended behaviour scale between Spanish and English population is very low, the difference in the authoritarianism subscale is very marked with far better attitudes for the English population. This difference between the Spanish and English populations could lie, as Busquets [44] describes, in the paternalistic mentality of health systems in Mediterranean countries.

In order to combat stigmatization, it has observed that public campaigns can be effective in achieving this specific goal [45, 46]. Arboleda-Flórez [47] pointed that there are some ways of fighting against authoritarianism such as education or contact based education where favourable outcomes are improving knowledge, attitudes, mental health literacy and reducing stereotypes and desire for interpersonal distance.

Factors that predict intended behaviour in the Spanish population are consistent with those in the English population with the exception of having or being in contact with a person with MD [22]. The only factor which has a similar impact on attitudes to MD among both the Spanish and English populations is the experience of being in contact with a person with MD. What is more, while gender impacts on attitudes of the English population, women have more favourable attitudes than men; in the Spanish population there is no difference in attitudes between genders, with the exception of the authoritarianism subscale. These differences between societies could be attributed to cultural differences. As this is the first study developed in Southern Europe, it would be desirable that this discussion be supported in other studies developed in the same population.

Rates of self-reported discrimination because of a MD are much lower than in studies in other countries such as Canada or the United States of America, where rates were reported in the range 38.5 to 47 % [48, 49] in comparison with a discrimination rate for the Catalan population of 3.8 % for those who were reported as having depression or anxiety and 15 % having another MD. However, our results are in line with other studies developed in European Union (EU) countries, which report rates of around 15 % [50]. The target populations in these studies and in our study were very similar. The differences among EU and North-American studies could be explained through differences in the conception of discrimination and/or differences in health system management. Another possible explanation could be that the Catalan population who had a MD would also have high rates of self-stigma and low rates of empowerment and, consequently, they are not aware that they are being discriminated against. [17]

Self-reported discrimination is higher in people who suffer MD other than depression or anxiety. As these other MDs are supposed to be more serious MDs, they could be associated with dangerous or violent behavior and be perceived as a more serious threat. This could be one reason why people with authoritarian mentalities display the least favourable attitude to people with MD.

Surprisingly, the fact of having a MD does not influence the majority of the attitudes about MD or intended behaviour. This is in contrast with a study in the United Kingdom where people that have a MD have more favourable attitudes and intended behaviour [22].In this scenario, we should take into account the self-stigma concept.

Consistent with Brusby Grant [46] and Latalova [51], between all the components of stigma, the one with the highest contribution is self-stigma, identifying it as an important barrier in the treatment of MDs and a crucial issue in the pathway to recovery. Unfortunately, this variable has not been evaluated.

On the other hand, being in contact with someone with a MD was associated with better attitudes and intended behaviour, as previously found by Evans-Lacko and colleagues [22]. This coincidence could be related to the implantation of social contact campaigns in both territories despite of the implementation of these campaigns in the United Kingdom are more developed [52].

The impact of being young in intended behaviour is even higher if it is modulated by having a MD or gender. Being male and younger are strengthen each other as it is shown in multivariate regression model without interactions while younger people with a MD seem to have a wider perspective regarding to mental health. This could be explained by living on a less stigmatizing society because the improvement in social policies.

### Strengths and Limitations

This study has used a large dataset which is representative of the Catalan population. Furthermore, to the best of our knowledge, this is the first study which evaluates stigma levels in a representative sample of the population in Southern Europe. Despite these strengths, we acknowledge some limitations linked to this study. First, the scales have limited metric properties for the population under study [30].We found many missing values in the CAMI scale, so we suggest that some of them are not completely understood by patients or the sample is impacted by item desirability. We imputed missing values through multiple imputations, trying to reduce this fact to the minimum. However, the wide use of these scales allows us to be able to compare the results of this study with others. Secondly, items come from an official survey which tends toward stiff and auto reported answers. Social desirability items could not be included in the survey, so this variable is not measured. In addition, interviewees report their own health disorders, so we are not certain about the presence of the condition. We must also emphasize that, for people who report a MD which is not depression or anxiety, we do not know the exact nature of their condition. However this is a common feature of this kind of study.

## Conclusions

Attitudes and intended behaviour toward mental disorders were favourable except for the authoritarianism attitudes subgroup. Accordingly, future actions addressed to decrease stigma in mental health should be pointed toward reducing the authoritarianism component of stigma.

This approach not only has to be followed for the general population but also for people who have had a MD.

Actions should also take into account not only depression and anxiety but other MDs where discrimination experiences are more common and authoritarianism seems to have more impact.

Public campaigns could be an important way to combat stigma in mental health. These campaigns should take into account the profile of the population in Southern Europe characterized by less favourable attitudes and intended behaviour. Campaigns could make more impact by targeting older people, immigrants, people with lower educational attainment and people who have not had contact with someone with a MD.

Future studies should explore the level of self-stigma and how it is related to identification of discrimination experiences, as well as how discrimination impacts on people with specific MDs other than depression and anxiety.

## Abbreviations

MDs: Mental Disorders
MD: mental disorder
CAMI: Community Attitude towards the Mentally Ill
DSS: Depression Stigma Scale
RIBS: Reported and Intended Behaviour Scale
ESCA: Enquesta de Salut de Catalunya
SCMH: support for community mental health care
EU: European Union
